# Barriers for return to work as an iatrogenic effect of sickness absence: a proposed conceptual framework and questionnaire based on a cross-sectional study

**DOI:** 10.1186/s12889-026-26584-1

**Published:** 2026-02-12

**Authors:** Malene Myhrer, Beate Brinchmann, Laurent Trichet, Nils Abel Aars, Arnstein Mykletun

**Affiliations:** 1 HelseIArbeid, Nordland Hospital Trust, Bodø, Norway; 2 Centre for Work and Mental Health, Nordland Hospital Trust, Bodø, Norway; 3 Department of Community Medicine, UiT- The Arctic University of Norway, Tromsø, Norway

**Keywords:** Sickness absence, Barriers, Return to work, Conceptual framework, Questionnaire

## Abstract

**Background:**

Sickness absence and disability incurs negative effects on both individuals and society as a whole. This necessitates more knowledge about the possible factors and mechanisms that perpetuate sickness absence, increasing the risk of permanent exclusion from the labour market. The aim of this study is to evaluate a proposed conceptual framework and questionnaire for barriers for return to work. Here, barriers are understood as iatrogenic effects of sickness absence that create a lock-in effect which impedes return to work and contributes to an exclusion trap.

**Methods:**

A questionnaire has been administered as part of a larger survey issued to patients prior to assessment in a Norwegian Sickness Absence Clinic (NSAC). We explored responses from individuals on sickness absence to the questionnaire, correlations between the questionnaire and work -and health-related measurement instruments, inter-item correlations and Cronbach’s alfa.

**Results:**

We observed associations between the barrier items and work -and health-related measurement instruments. The questionnaire demonstrates a strong internal consistency, with a Cronbach’s Alfa of 0.83. Results indicate a need for revision of the questionnaire.

**Conclusions:**

Patients on sickness absence reported several barriers for return to work. The most prevalent barriers, reflecting cognitions about health, agreed with our proposed conceptual framework of barriers for return to work as iatrogenic effects of sickness absence. Exploring patients’ cognitions in the return-to-work process and intervening on them through well-established intervention strategies like graded exposure and belief change, could be of potential importance in reducing detrimental side effects of sickness absence.

**Trial registration:**

The trial was prospective registered at clinicaltrials.gov on August 9th, 2021. Trial identifier NCT 05006976.

**Supplementary Information:**

The online version contains supplementary material available at 10.1186/s12889-026-26584-1.

## Background

High levels of sickness absence (SA) and disability benefit pose a challenge to the sustainability of the workforce in public sector and the welfare state in many OECD countries. Public spending on sickness and disability benefits constitutes approximately 2% of GDP on average, exceeding 4% in countries like Norway and Denmark [1]. Beyond the detrimental consequences to society, worklessness is linked to individual health disparities [2], and the probability of returning to work (RTW) declines markedly the longer individuals are away from work [3, 4]. Improving health is not in itself sufficient for successful RTW [5–7]. This indicates that, on par with reduced symptoms, factors arise during SA that negatively affect RTW, in turn contributing to an exclusion trap through prolonged work absence and increased probability of permanent exit from the labour force. Such an iatrogenic effect of SA could be overlooked in clinicians’ drive to provide patients with support, relief and cure.

There is a substantial body of research on barriers for RTW that includes both individual, social, environmental, judicial and structural factors. Furthermore, instruments have been developed to identify and intervene on barriers for RTW [8, 9]. Evidence suggests that interventions focused on identifying and modifying barriers positively impacts RTW [10, 11]. Existing literature is, however, unclear about what constitutes a barrier (internal versus external to the individual, modifiable versus non-modifiable) and, more importantly, the mechanisms of which barriers affect RTW.

We propose a conceptual framework wherein barriers for RTW are defined as *“cognitive*,* relational and/or emotional iatrogenic effects of sickness absence experienced by the individual”.* We have developed a questionnaire as a measurement instrument for barriers for RTW, in line with our proposed framework. Within this framework, barriers are thought to manifest as a function of absence from work, creating a lock-in effect that impedes RTW. This could help explain the inverse relationship between duration of SA and RTW, drawing parallels to how avoidance behaviour perpetuates and worsens health and functioning.

This mechanism can be elucidated through the example of anxiety, wherein individuals typically develop maladaptive strategies (such as avoidance of the feared stimuli) and negative beliefs (such as believing oneself to be socially undesirable). Treatment focused on exposure to the feared stimuli and modifying negative beliefs, relieves anxiety [12]. Similarly, SA can reflect an artificial avoidance that can contribute to negative beliefs (i.e.: barriers for RTW) that perpetuate SA, making exposure to work and changing barrier beliefs potentially potent interventions in reducing long-term SA. This conceptual framework concurs with research showing graded SA to be more beneficial for RTW than full SA [6, 13]. An important cognitive change highlighted as a probable mechanism in exposure therapy is change in self-efficacy [14]. In addition to possibly changing barrier beliefs, work exposure can improve self-efficacy [15], which in turn predicts RTW [16].

The aim of this article is to assess a conceptual framework and questionnaire of barriers for RTW, with the aim of developing a clearly defined way of understanding, measuring and intervening on barriers for RTW. In this article we will evaluate (1) how common barriers for RTW are, (2) whether the proposed barriers of the questionnaire are novel, and (3) whether the questionnaire needs revision.

The results will guide the development of the conceptual framework and questionnaire. This, in turn, can guide interventions and techniques which aim to reduce the probability of a SA-incurred exclusion trap, benefiting not only the individual, but also the workplace and society as a whole.

## Methods

### Development of a conceptual framework and questionnaire

The conceptual formulation and development of the barrier questionnaire involved an expert panel of 15 clinicians and researchers in the field of work and health in Norway, England and Australia. The clinicians, comprising most of the group, were psychologists, psychiatrists and physiotherapists. The conceptual framework and questionnaire were developed iteratively over a period of 12 years, informed by expert discussions of anonymised cases from clinical practice and a theoretical framework integrating cognitive theory, social psychology and behavioural economics, without following a formally standardised development protocol. The barrier questionnaire consists of 26 individual items scored on a Likert scale from 1 (disagree) to 5 (agree), which is further divided into five subgroups; Family obligations, Emotions about RTW, Cognitions about health, Assessment of health problems and Work-related factors (see Table 1 for an overview). The questionnaire has been published previously in the protocol for the trial [17].

**Table 1:** Barriers for return to work. Likert scale from 1 (disagree) to 5 (agree)

There can be many barriers that prevent you from returning to work when you are on sick leave. How do you perceive the following statements?	1	2	3	4	5
*Family Obligations*					
I can’t go back to work now because I’m responsible for the care of a friend or family member					
I can’t go back to work now because I’m in a stressful life situation (for example, illness of others, breakup, conflict or death in my own family)					
I can’t go back to work now because I don’t have enough time					
*Emotions about RTW*					
I can’t go back to work now because I don’t feel ready to start working yet					
I can’t go back to work now because I don’t master my job					
I can’t go back to work now because I’m not motivated for the job I have now					
I can’t go back to work now because I have to set boundaries for myself					
I can’t go back to work now because I can then be laid off, downsized or relocated					
*Cognitions about health*					
I can’t go back to work now because I have to get well first					
I can’t go back to work now because I can get sicker if I go back to work					
I can’t go back to work now because my doctor thinks I should be on sick leave now					
I can’t go back to work now because I have to focus on treatment					
*Assessment of health problems*					
I can’t go back to work now because I have too much physical pain*					
I can’t go back to work now because I have concentration problems*					
I can’t go back to work now because I’m too anxious or scared*					
I can’t go back to work now because I’m too depressed*					
I can’t go back to work now because I do not have enough energy*					
I can’t go back to work now because I have too much sleep problems*					
*Work-related factors*					
I can’t go back to work now because my employer does not adapt tasks to my situation					
I can’t go back to work now because I am being treated badly at work*					
I can’t go back to work now because I am not wanted at work					
I can’t go back to work now because I’m in a conflict at work					
I can’t go back to work now because I’m waiting for an apology					
I can’t go back to work now because I’m trying to send a signal to management or the business					
I can’t go back to work now because something at work has changed while I have been on sick leave					
I can’t go back to work now because the job makes me sick					

Name of subgroup written in cursive. The subgroups are not presented to patients when answering the questionnaire. Items marked with an asterisk are proposed removed or relocated in a revised version of the questionnaire. Correlations between items of the subgroup *Assessment of health problems* with other symptom questionnaires suggests removal of these items. “I have too much physical pain” could be relocated to the subgroup *Cognitions about health*. Furthermore, based on inter-item correlations, we suggest removing the item “I am being treated badly at work”

### Study setting and data collection

**Fig. 1:**
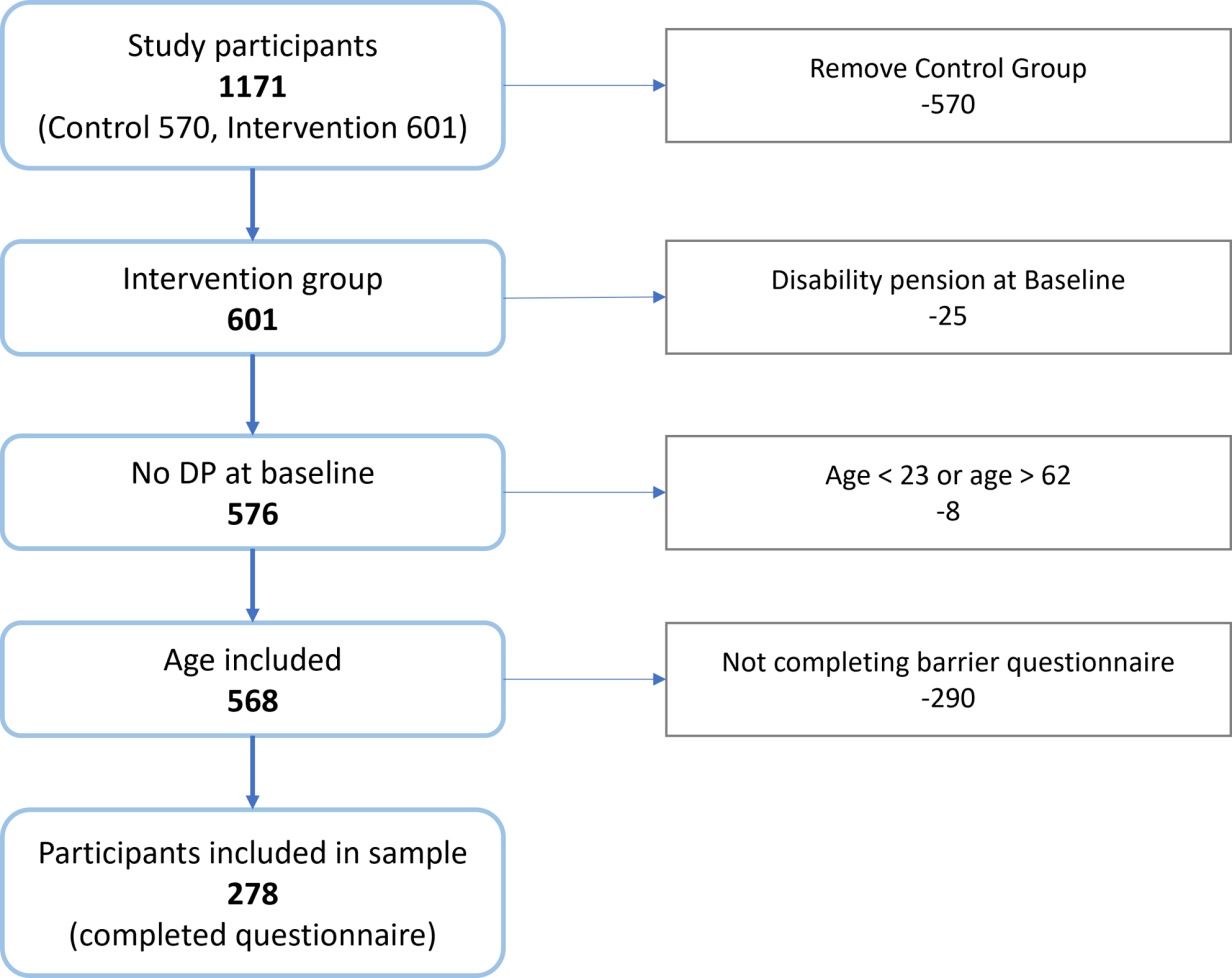
Flow diagram illustrating the selection process

The current study is based on secondary use of data collected as part of a multicentre randomised controlled trial [17], led by the Centre for Work and Mental Health at Nordland Hospital, Bodø, Norway. The trial recruited 1171 patients from five Norwegian Sickness Absence Clinics (NSACs) in Northern Norway over a period of 16 months, from September 6th 2021 to January 15th 2023. The clinics offer coordinated, interdisciplinary evaluation and treatment of patients referred from their general practitioner (GP), aiming to aid the patient in maintaining or returning to work/studies and managing health problems. The target group is people at risk of, or on, sick leave due to common mental health disorders (in this study defined as mild to moderate anxiety and depression) and/or musculoskeletal disorders. Treatment eligibility is evaluated by an interdisciplinary team consisting of a physician, psychologist and physiotherapist. Patients that are too healthy, too sick, do not have a relevant diagnosis or are otherwise not considered to be in the target group, are not eligible for assessment in the clinic. In this case, too sick encompasses more severe mental health disorders such as bipolar disorder and severe depression, or individuals with severely impaired functioning. Conversely, too healthy implies that the patient was considered too well to require specialist healthcare and should instead receive follow-up in primary care, or that the patient presented with conditions or reactions deemed normal and/or transient (such as grief). Before the first consultation patients were asked to fill out a comprehensive online questionnaire, called baseline survey, sent by electronic mail. The control group received a questionnaire mapping health factors only, whereas the questionnaire issued to the intervention group also included work-related topics pertaining to Motivation, Barriers for RTW and Work environment (MBW). Given the aim of the present study, the following participants were excluded from our analyses; (1) patients not randomised to the intervention group, (2) recipients of disability pension at baseline, (3) patients aged < 23 years old or > 62 years old, and (4) patients not completing the questionnaire. The selection process is displayed in Fig. 1.

### Study design and statistical analyses

First, we explored the prevalence of individual barriers for RTW by looking at individual responses to the items of the questionnaire. Second, we evaluated whether the barriers of the questionnaire contributed to something new by correlating the individual barrier items with other instruments measuring work and health-related factors. Finally, we evaluated the need for revision by conducting inter-item correlations, Cronbach’s alfa for internal consistency and correlations with other instruments from the survey.

Sample characteristics at baseline were analysed by descriptive statistics. Inter-item correlations and correlations between barrier items and other scales were assessed by the Pearson method. Interpretation of correlation coefficients were done in accordance with Dancey and Reidy [18], categorising 0.0 to 0.3 as weak, 0.4 to 0.6 as moderate and 0.7 to 1.0 as strong correlations. Internal consistency of the 26 items of the barrier questionnaire were assessed using Cronbach’s alpha coefficient. A value of α ≥ 0.70 was considered acceptable for reliability, and a value of α ≥ 0.80 was considered preferable [19]. We considered *p* < 0.05 to be of statistical significance. Cronbach’s alfa was also estimated on deletion of each of the 26 items. Data management and statistical analysis were performed using Python version 3.8 (Python Software Foundation) for correlations, and IBM SPSS Statistics for Windows, Version 29.0 (IBM Corp. Armonk, NY) for descriptive statistics, frequency distributions, and internal consistency.

### Data

Other than the variables included in the barrier questionnaire (outlined in Table 1), we conducted correlations between the barrier questionnaire and health and work-related measurement instruments. For a complete overview and description of the measurement instruments included, we refer to the protocol paper [17]. Procedural justice and relational justice were both assessed as components of organisational justice in Kivimäki et al. [20]. While procedural justice pertains to fairness in decision making procedures (such as hearing the opinions of all affected parties before making a decision), relational justice measures how individuals are treated by their supervisor. Furthermore, the Demand Control Support Questionnaire (DCSQ) was separated into the means of items measuring perceived support from colleagues and supervisors (items 12 to 17) and items measuring demand and control (items 1 to 10). Similarly, the means of items within the subgroup Physical Activity in Fear Avoidance Beliefs Questionnaire (FABQ) were separated from the means of items within the subgroup Work in the correlational analyses. For Effort Reward Imbalance (ERI) we correlated our barrier questionnaire with the group of items measuring effort (3 items) and reward (7 items), respectively.

## Results

### Sample characteristics

The sample characteristics of the included sample is shown in Table 2. Overall, 278 patients met the inclusion criteria for the present study. Most of the participants were women (70.1%). The mean age was 42.4 years (SD = 10.3), ranging from 23 to 62 years of age. A marginal majority of the participants had completed higher education (54.1%), whereas the rest had completed primary school (9.1%) and high school (36.9%). Participants married, cohabitant or in a partnership constituted 74.2% of the sample.

**Table 2:** Sample characteristics

Sex	*N* (%)
Female	195 (70.1)
Male	83 (29.9)
Age	
23–29 years	39 (14.0)
30–39 years	76 (27.3)
40–49 years	85 (30.6)
50–62 years	78 (28.1)
Mean	42.4 (10.3)
Marital status	
Married, cohabitant or partner	204 (74.2)
Single	53 (19.3)
Widow/widower	2 (0.7)
Separated/divorced	16 (5.8)
Educational level	
Primary school	25 (9.1)
High school	101 (36.9)
University up to 3 years	84 (30.7)
University over 4 years	64 (23.4)

Distribution of responses to the questionnaire

Most barriers for RTW were low-prevalent, while some were universal to the respondents (additional file 1). The items with overall highest responses were “I can get sicker if I go back to work” (M = 3.85), “I do not have enough energy” (3.65), “I have to get well first” (M = 3.55), and “I don’t feel ready to start working yet” (M = 3.55). Most of the patients reported multiple barriers for RTW, responding 3 or higher on 10.3 barriers items. Most of the barrier items were, however, of low prevalence, especially “I’m waiting for an apology” (M = 1.11), “I’m trying to send a signal to management or the business” (M = 1.18), “something at work has changed while I have been on sick leave” (M = 1.22), and “I can then be laid off, downsized or relocated” (M = 1.22). Although most of the barrier items show low prevalence, a small percentage reported these items as strong barriers for RTW. This pattern cannot be explained by certain individuals responding 5 on every item of the questionnaire, seeing as only 9 of the 278 participants responded 5 on more than 10 items.

### Inter-item correlations

The inter-item correlations are available in additional file 2. Correlations within subgroups of barriers were positive, except for “I have too much physical pain”. Within the subgroup *Emotions about RTW*, the item “I can then be laid off, downsized or relocated” was weakly correlated to all items of the questionnaire, relatively highest correlations being with the items “I am not wanted at work”, and “something at work has changed while I have been on sick leave”. The item “I’m not motivated for the job I have now” correlated moderately with the items “I’m too depressed”, “I’m too anxious or scared”, and “I am being treated badly at work”.

Items in *Cognitions about health* correlated relatively weak with other items of the barrier questionnaire, except moderate correlations between “I can get sicker if I go back to work”, and “I don’t feel ready to start working yet” and “the job makes me sick”, respectively.

In the subgroup *Assessment of health problems*, all items but one correlated positively with remaining items of the questionnaire. The weakest correlations were with items of the subgroup *Cognitions about health.* “I have too much physical pain”, correlated negatively with all items except “I have too much sleep problems” and items of the subgroup *Cognitions about health.*

*Work-related factors* had strong inter-item correlations between “I am being treated badly at work”, “I am not wanted at work”, and “I’m in a conflict at work”. With the exception of two aforementioned items, correlations between items in *Work-related factors* and remaining items of the questionnaire were weak. “I’m trying to send a signal to management or the business”, and “I’m waiting for an apology” had the overall weakest correlations.

### Internal consistency

The barrier questionnaire had a Cronbach’s Alfa of 0.83, indicating a good internal consistency (IC). Upon removal of single items, IC remained between 0.82 and 0.85, suggesting that none of the items were problematic for the IC. The only item that increased IC slightly when removed was “I have too much physical pain”.

### Correlations between barrier items and other instruments

Correlations between barrier items and other instruments is available in additional file 3. *Family obligations* had a positive, moderate correlation with Work-Home-Interference (WHI), suggesting similarities but not complete overlap. WHI also had positive, but weaker correlations with the subgroups *Emotions about RTW*, *Assessment of health problems* and Work-related factors. 

Beck Depression Inventory (BDI), Beck Anxiety Inventory (BAI), Hopkins Symptoms Checklist 10 (HSCL-10) and Fatigue Assessment Scale (FAS) had positive, moderate correlations with items of the subgroup *Assessment of health problems*, with stronger correlations for barrier items of special relevance to the questionnaire (such as “I’m too anxious or scared” and BAI, and “I do not have enough energy” and FAS).

“I have too much physical pain”, the last item of the subgroup *Assessment of health problems*, correlated weakly with BAI, BDI, HSCL-10 and FAS, but showed moderate correlations with Oswestry Disability Index (ODI) and Fear Avoidance Beliefs Questionnaire for Work (FABQ-W). Uniformly, ODI and FABQ-W correlated weakly with items of the subgroup *Assessment of health problems*, and comparatively stronger with *Cognitions about health*, especially “I have to get well first”, “I have to focus on treatment”, and “I can get sicker if I go back to work”. The subgroup *Cognitions about health* had relatively strongest correlations with Sick Leave As Prevention (SLAP).

Correlations between the whole barrier questionnaire, and the subgroup *Work-related factors* specifically, with work-related measurement instruments, were mostly weak, except moderate correlations between Closest manager, Short Negative Act Questionnaire (SNAQ), the support part of the Demand Control Support Questionnaire (DCSQ), relational justice and reward as measured by items in ERI. The highest correlations for both the work subgroup and the total of the barrier questionnaire were found with Job Phobia.

## Discussion

In this cross-sectional study of 278 sick-listed patients admitted to secondary care assessment, our proposed concept for barriers for RTW appears to be of significant importance. While most items occur seldom they are still perceived by some individuals as strong barriers for RTW. Correlations with other measurement instruments indicate expected overlap, especially pertaining to work -and health-related factors. Some barriers for RTW, however, seem more novel. These barriers for RTW, being the most prevalent, could be understood as maladaptive cognitions and fear-avoidance behaviour that perpetuate SA.

The most prevalent barriers for RTW are found in the subgroup *Cognitions about health*. The item “I can get sicker if I go back to work” is in line with the fear of relapse-dimension included in Return-to-Work Obstacles and Self-Efficacy Scale (ROSES), and is documented in prior barrier research [21–23]. Conversely, “I have to get well first” and “I have to focus on treatment”, do not seem to overlap with existing barriers for RTW described in ROSES or Obstacles to Return-to-Work Questionnaire (ORQ) [8, 9], and is scarcely mentioned in barrier research [23, 24]. Similarities for the last item, “My doctor thinks I should be on sick leave now”, can be found in barrier research, described as the system not facilitating RTW [21], and as a lack of coordination between systems [22, 23]. This system-focused understanding diverges with ours, in that our concept does not measure what the doctor’s thoughts or recommendations are regarding the patient’s SA, but rather what the patient assumes or perceives their doctor to think.

These highly prevalent and seemingly more novel barrier items seem to overlap with barriers understood in the literature as illness representations [23–25], in line with Leventhal`s Common Sense Model of Illness Representations [26]. Both the Common Sense Model of Illness Representations and the Health Belief Model [27] posit that the individual has cognitions about one’s symptom/illness, and that these cognitions guide behaviour expected to be beneficial to health. Analysis indicates that some variables of the health belief model are predictive of health-related behaviour, especially perceived barriers and benefits to performing the behaviour [28]. Furthermore, illness perceptions have been associated with work participation [29, 30].

Understanding the barriers of the subgroup *Cognitions about health* within the framework of these models, it can be argued that the individual’s cognitions about their symptoms/illness (including perceived consequences, such as impact on work capacity) can lead to beliefs about SA being the most beneficial course of action for reducing health threat or improving health. More specifically, if a sick-listed individual with low back pain associates work with pain, believes that RTW will worsen pain, and thinks SA will be more beneficial in reducing pain, then they will seek to maintain SA. This aligns with our conceptual framework, proposing that absence from work and emergence of barriers perpetuate SA in the same manner as avoidance and maladaptive beliefs maintain anxiety.

Although a large multilevel analysis indicated that 98% of the unexplained variation in long-term SA is explained by patient factors [31], GPs can have a potentially large impact on individuals’ cognitions about their health and how to manage their illness. Employees report more negative illness perceptions than their occupational physicians, and they more often associate their illness with work [30]. Furthermore, GPs’ fear avoidance beliefs influence their actions and recommendations regarding occupational and physical activities for patients with low back pain [32]. Findings indicate that patient`s perceptions of pain and disability correlate with practitioner`s decisions regarding sick leave [33]. If this is due to GPs largely playing an advocate role for their patients, emphasising their wishes when making decisions [31], it may perpetuate and worsen the individual’s cognitions and actions regarding health and work.

Results seem to indicate that the item “I have too much physical pain” reflects perceived disability and fear of RTW leading to increased pain, more than pain intensity per se. This is in line with the fear-avoidance model of chronic pain, which posits that pain-related fear leads to maladaptive avoidance-strategies which maintain fear and disability over time [34]. Maladaptive avoidance-strategies are associated with threat perceptions and emotion representations measured within the framework of the Common Sense Model of Illness Representations [35], and high degree of fear-avoidance beliefs predicts work-related outcomes [36, 37].

Another subgroup of barriers for RTW showing high prevalence amongst individuals on SA, is *Emotions about RTW*. Two of the items could be understood as motivation for work and as RTW-self-efficacy (RTW-SE), both of which are associated with RTW outcomes [5, 25, 38]. The remaining items of the subgroup could, however, be argued to offer a more novel contribution to the barrier literature. These items could reflect a state or mode of individuals on SA. Research has indicated that individuals seem to have different attitudes towards RTW, where many remain passive in implementing solutions that can aid in the RTW-process, reflecting an intention-behaviour gap [22, 39, 40]. Given the moderate correlations between items of this subgroup with the Job Phobia Questionnaire, this intention-behaviour gap could arguably be driven by fear avoidance.

Items of the subgroup *Assessment of health problems* overlap with symptom checklists such as HSCL-10, BAI, BDI and FAS. Results are convergent with existing literature identifying anxiety, depression, concentration issues and fatigue as barriers for RTW [5, 16, 24, 25, 41], and is included in other barrier instruments [8, 9]. Although symptom reduction is beneficial for RTW [42], inter alia through increased RTW-SE [5, 41], it is insufficient [5–7]. This could be explained by the presence of other barriers for RTW that need to be addressed through other means than symptom reduction, in line with conclusions from Audhoe and colleagues [39] and further supported by findings showing work-focused CBT to be more effective than standard CBT for RTW [6].

Although many barriers for RTW are less prevalent, they are perceived as a strong barrier by some. The impact of one strongly perceived barrier for RTW compared to the cumulative impact of multiple barriers is not known regarding RTW, and importantly a low prevalence of an item is not necessarily equal to low importance with respect to RTW. An example is the item “I am not wanted at work”, which is moderately correlated with bullying, as measured by SNAQ. Bullying can be detrimental to health and work ability [43] and is associated with RTW [7]. Similarly, perceived social support, arguably measured by items of the subgroup *Work-related factors*, is important both before, during and after RTW [22]. High inter-item correlations between three items measuring perceived social support at work, could indicate possible redundancy. See Table 1 for a proposed revision of the questionnaire.

Given the importance of perceived social support for RTW [22, 44, 45], efforts to improve support and understanding for the sick-listed individual both at work and at home could be of significance. One study found that patients on graded SA expressed the highest agreement with a statement that their employer would like them back at work [33]. One explanation, as argued by the authors, is that a well organised graded SA and work arrangement foster positive workplace beliefs and relationships. It could, however, also be that graded work exposure challenges some of the beliefs that otherwise could arise as a consequence of absence from work, as proposed by our conceptual framework.

Our results seem to indicate that maladaptive cognitions and fear-avoidance behaviour could be central in perpetuating SA. Interventions aimed at changing maladaptive cognitions, using methods such as cognitive behavioural therapy, metacognitive therapy and exposure therapy, could be of great importance in reducing avoidant coping behaviour and increasing probability of RTW, in line with the conclusions from Hoving and colleagues [29]. Reducing fear-avoidance beliefs has been associated with improved disability outcomes [37]. Work exposure through graded SA would also be considered beneficial as a means of challenging avoidance behaviour and maladaptive beliefs [46]. Given the observed intention-behaviour gap of individuals on SA regarding RTW, focusing on developing a RTW plan with the patient will also help improve RTW outcomes [5].

The iatrogenic effects of SA through emergence of barriers for RTW should not necessarily be addressed and intervened on as part of individual treatment alone, as the concept is also of great importance for those with a role in the support or care of the sick-listed individual. Illness perceptions of significant others are associated with duration of SA, indicating a need to involve them in clinical interventions [47]. Furthermore, although GPs emphasise patient’s wishes when making decisions [31] and rarely overrule patient requests for SA [48], they have an ethical obligation to first do no harm (primum non nocere). If SA brings with it iatrogenic effects that increase the risk of permanent exclusion from the labour force, the potential long-term harms must be considered carefully. Exploring patient’s arguments for and beliefs about SA, and presenting and managing SA like other medical treatments (with possible effects and side effects) that needs to be evaluated continuously, can help identify and address maladaptive beliefs and iatrogenic effects together with the patient. This, in turn, could potentially improve speed and rate of RTW, avoiding the lock-in effect and potential exclusion trap of perpetuated SA.

Although interventions at the individual level are important, efforts to improve RTW and reduce absence from the labour force should also be facilitated at the systems -and political level. Norway has the highest rate of SA amongst OECD-countries and a generous sick-leave practice with few economic incentives for RTW for both the sick-listed individual, the GP and the employer [1, 48, 49]. Arguably, such a system could contribute to further maintaining and worsen the iatrogenic effects of SA by enabling maladaptive avoidance-behaviour and beliefs to affect decisions regarding work, and by not presenting effective means of bridging the intention-behaviour gap perpetuating SA. In this regard, future research should explore potential differences in iatrogenic effects of SA in countries with different SA-practices and legislations.

The potential implications of the conceptual framework and questionnaire are, however, dependent upon feedback from clinicians’ regarding the usefulness and utility of it, as well as its potential effects in reducing SA. In one study [50], nudging patients and clinicians on MBW-factors, herein barriers for RTW, did not have any effect on RTW compared to a group receiving only health-related questionnaires. The authors did, however, acknowledge the limitation that the clinicians were already well versed in delivering work-focused treatment, making the nudge redundant and hence ineffective. Furthermore, research on possible causal iatrogenic effects of SA is needed, differentiating between factors present before or at onset of SA and factors arising during SA, to further evaluate the theoretical soundness and empirical validity of our proposed framework.

### Limitations and future directions

The strength of this study is that it is part of a multi-centre randomised controlled trial. Patients from different locations contribute to heterogeneity of the sample. Given the extensiveness of the questionnaire, it is possible that patients more severely affected by physical, mental or cognitive issues were excluded from the sample. Furthermore, the conceptual framework and developed questionnaire for barriers for RTW is new and therefore not validated. This article is an attempt to remedy this. Furthermore, the predictive validity of the questionnaire is yet to be known. This should be explored in further research and will help guide decisions regarding revision of the questionnaire. Further research should also aim to differentiate between graded and full SA, as well as exploring the effects of duration of SA, given that these are reported to affect SA and RTW. There is also a need for further research to evaluate the clinical utility of the proposed conceptual framework and barrier questionnaire.

## Conclusions

In this article, we propose that iatrogenic effects of SA can create a lock-in effect that may contribute to permanent exclusion from the labour market. This could help explain why symptom reduction is not sufficient for RTW, and why graded SA is more effective for RTW than full SA. To better understand these iatrogenic effects, we have developed a questionnaire to measure perceived barriers for RTW. Evaluating our proposed conceptual framework and barrier questionnaire is important to illuminate weather this could be an important contribution to the research field and clinical practice, possibly informing further research and clinical interventions. Several barriers for RTW are reported by patients on SA. Other barriers, however, seem less frequent, but are nonetheless reported as strong barriers for RTW by some individuals. Most of the items of the barrier questionnaire are convergent with existing research and theories, while some of the items seem to be important contributions to the field. This especially pertains to cognitions and actions driven by fear-avoidance and illness perceptions. In line with our conceptual framework, our findings indicate that solely focusing on symptom reduction or work environment will not effectively alter perceived barriers for RTW. Interventions aimed at modifying the individuals’ cognitions and actions regarding RTW can, however, be of great importance in reducing the individual and societal consequences of SA. Such interventions, including exposure therapy and belief change, can be easily integrated in existing treatment and intervention programs. Results indicate possible redundancy of some items. Analysis of predictive value of the barriers for RTW, as well as clinicians’ assessment of relevancy and value of the different items, will aid in further revision of the barrier questionnaire.

## Supplementary Information

Additional file 1. Response distribution to barrier items. Overview of barrier items, with response distribution, histogram, mean and standard deviation of each item.

Additional file 2. Inter-item correlations of the barrier questionnaire. Tabular overview of correlations between all items of the questionnaire.

Additional file 3. Correlations between the barrier questionnaire and other questionnaires. The file presents correlations between single items, subgroups of items and the mean of the barrier questionnaire, with the means of measurement instruments for health and work.

## Data Availability

The datasets analysed during the current study are available from the authors on reasonable request.

## Abbreviations

SA: Sickness absence
RTW: Return to work
HSCL-10: Hopkins Symptom Checklist 10
BAI: Beck Anxiety Inventory
BDI: Beck Depression Inventory
FAS: Fatigue Assessment Scale
WI: Whiteley Index
NDI: Neck Disability Index
ODI: Oswestry Disability Index
FABQ-P: Fear Avoidance Beliefs Questionnaire, Physical Activity subscale
FABQ-W: Fear Avoidance Beliefs Questionnaire, Work subscale
WHI: Work-Home Interference
ERI: Effort-Reward Imbalance
DCSQ (dc): demand control support questionnaire (items measuring demand and control)
DCSQ (s): demand control support questionnaire (items measuring support)
SNAQ: Short Negative Act Questionnaire
SLAP: Sick Leave as Prevention

## References

[1] Economic Surveys OECDOECD. Norway 2024. Paris: OECD Publishing; 2024. [cited 2024 Dec 12]. 10.1787/cb13475f-en.

[2] Modini M, Joyce S, Mykletun A, Christensen H, Bryant EA, Mitchell PB, Harvey SB. The mental health benefits of employment: results of a systematic meta-review. Australas Psychiatry. 2016;24:331–6. 10.1177/1039856215618523. 26773063 10.1177/1039856215618523

[3] OECD, Fit Mind F, Job. From Evidence to Practice in Mental Health and Work. Paris: OECD Publishing. 2015. Available from: 10.1787/9789264228283-en. Cited 2025 Jan 21.

[4] van Hoffen MFA, Joling CI, Heymans MW, Twisk JWR, Roelen CAM. Mental health symptoms identify workers at risk of long-term sickness absence due to mental disorders: prospective cohort study with 2-year follow-up. BMC Public Health. 2015;151235. 10.1186/s12889-015-2580-x. 10.1186/s12889-015-2580-x PMC4676883 26655203

[5] Cancelliere C, Donovan J, Stochkendahl MJ, Biscardi M, Ammendolia C, Myburgh C, Cassidy JD. Factors affecting return to work after injury or illness: best evidence synthesis of systematic reviews. Chiropr Man Th. 2016;24:32. 10.1186/s12998-016-0113-z. 10.1186/s12998-016-0113-z PMC5015229 27610218

[6] Cullen KL, Irvin E, Collie A, Clay F, Gensby U, Jennings PA, et al. Effectiveness of workplace interventions in return-to-work for musculoskeletal, pain-related and mental health conditions: an update of the evidence and messages for practitioners. J Occup Rehabil. 2018;28:1–15. 10.1007/s10926-016-9690-x. 28224415 10.1007/s10926-016-9690-x PMC5820404

[7] de Vries H, Fishta A, Weikert B, Sanchez AR, Wegewitz U. Determinants of sickness absence and return to work among employees with common mental disorders: a scoping review. J Occup Rehabil. 2018;28:393–417. 10.1007/s10926-017-9730-1. 28980107 10.1007/s10926-017-9730-1 PMC6096498

[8] Corbière M, Negrini A, Durand MJ, et al. Development of the Return-to-Work Obstacles and Self-Efficacy scale (ROSES) and validation with workers suffering from a common mental disorder or musculoskeletal disorder. J Occup Rehabil. 2017;27:329–41. 10.1007/s10926-016-9661-2. 27562583 10.1007/s10926-016-9661-2

[9] Marhold C, Linton SJ, Melin L. Identification of Obstacles for chronic pain patients to return to work: evaluation of a questionnaire. J Occup Rehabil. 2002;12:65–75. 10.1023/A:1015056429505. 12014227 10.1023/a:1015056429505

[10] Marois E, Durand MJ. Does participation in an interdisciplinary work rehabilitation programme influence return-to-work Obstacles and predictive factors? Disabil Rehabil. 2009;31:994–1007. 10.1080/09638280802428374. 19037769 10.1080/09638280802428374

[11] Dol M, Varatharajan S, Neiterman E, McKnight E, Crouch M, McDonald E, et al. Systematic review of the impact on return to work of return-to-work coordinators. J Occup Rehabil. 2021;31:675–98. 10.1007/s10926-021-09975-6. 33881671 10.1007/s10926-021-09975-6

[12] Knowles KA, Tolin DF. Mechanisms of action in exposure therapy. Curr Psychiatry Rep. 2022;24:861–9. 10.1007/s11920-022-01391-8. 36399234 10.1007/s11920-022-01391-8

[13] Bardal I, Aars NAP, Sanatkar S, Stevelink SAM, Brandseth OL, Brinchmann B, Mykletun A. Is the association between graded sickness absence and return to work confounded by health? A longitudinal cohort study from the Norwegian neck and back registry. BMC Public Health. 2025;25:1202. 10.1186/s12889-025-22368-1. 40159467 10.1186/s12889-025-22368-1 PMC11956489

[14] Breuninger C, Tuschen-Caffier B, Svaldi J. Dysfunctional cognition and self-efficacy as mediators of symptom change in exposure therapy for agoraphobia: systematic review and meta-analysis. Behav Res Ther. 2019;120:103443. 10.1016/j.brat.2019.103443. 31374484 10.1016/j.brat.2019.103443

[15] Lagerveld SE, Brenninkmeijer V, Blonk RWB, Twisk JWR, Schaufeli WB. Predictive value of work-related self-efficacy change on return to work for employees with common mental disorders. J Occup Environ Med. 2017;74:381–3. 10.1136/oemed-2016-104039. 10.1136/oemed-2016-104039 28007760

[16] Fisker J, Hjorthøj C, Hellström L, Mundy SS, Rosenberg NG, Eplov LF. Predictors of return to work for people on sick leave with common mental disorders: a systematic review and meta-analysis. Int Arch Occup Environ Health. 2022;95:1–13. 10.1007/s00420-021-01827-3. 35106629 10.1007/s00420-021-01827-3

[17] Aars NA, Bardal I, Brinchmann B, Mykletun A. Naturalistic trial of nudging patients and clinicians in the Norwegian sickness absence clinics: a study protocol for the NSAC nudge study. BMJ Open. 2025;15(3):e089758. 10.1136/bmjopen-2024-089758. 40032398 10.1136/bmjopen-2024-089758 PMC11877198

[18] Akoglu H. User’s guide to correlation coefficients. Turk J Emerg Med. 2018;18(3):91–3. 10.1016/j.tjem.2018.08.001. 30191186 10.1016/j.tjem.2018.08.001 PMC6107969

[19] Pallant J. SPSS survival manual: A step by step guide to data analysis using IBM SPSS. 7th ed. London: Routledge; 2020.

[20] Kivimäki M, Elovainio M, Vahtera J, Ferrie JE. Organisational justice and health of employees: prospective cohort study. Occup Environ Med. 2003;60:27–34. 12499453 10.1136/oem.60.1.27 PMC1740369

[21] Toye F, Seers K, Allcock N, Briggs M, Carr E, Barker K. A synthesis of qualitative research exploring the barriers to staying in work with chronic musculoskeletal pain. Disabil Rehabil. 2016;38(6):566–72. 10.3109/09638288.2015.1049377. 26017361 10.3109/09638288.2015.1049377

[22] Andersen MF, Nielsen KM, Brinkmann S. Meta-synthesis of qualitative research on return to work among employees with common mental disorders. Scand J Work Environ Health. 2012;38(2):93–104. http://www.jstor.org/stable/41508872. 22025244 10.5271/sjweh.3257

[23] Dekkers-Sánchez PM, Wind H, Frings-Dresen MHW, Sluiter JK. Implementation of a checklist to assess factors relevant for work ability assessments of employees on long-term sick leave. Int Arch Occup Environ Health. 2015;88:577–88. 10.1007/s00420-014-0975-0. 25252737 10.1007/s00420-014-0975-0

[24] Dekkers-Sánchez P, Wind H, Sluiter JK, Frings-Dresen MHW. A qualitative study of perpetuating factors for long-term sick leave and promoting factors for return to work: chronic work disabled patients in their own words. J Rehabil Med. 2010;42:544–52. 20549159 10.2340/16501977-0544

[25] Gragnano A, Negrini A, Miglioretti M, Corbière M. Common psychosocial factors predicting return to work after common mental disorders, cardiovascular diseases, and cancers: a review of reviews supporting a cross-disease approach. J Occup Rehabil. 2018;28:215–31. 10.1007/s10926-017-9714-1. 28589524 10.1007/s10926-017-9714-1

[26] Leventhal H, Meyer D, Nerenz D. The common sense model of illness danger. In: Rachman S, editor. Medical psychology. Volume 2. New York: Pergamon; 1980. pp. 7–30.

[27] Strecher VJ, Rosenstock IM. The health belief model. In: Baum A, Newman S, Weinman J, West R, McManus C, editors. Cambridge handbook of Psychology, health and medicine. Cambridge: Cambridge University Press; 1997.

[28] Carpenter CJ. A meta-analysis of the effectiveness of health belief model variables in predicting behavior. J Health Commun. 2010;25(8):661–9. 10.1080/10410236.2010.521906. 10.1080/10410236.2010.521906 21153982

[29] Hoving JL, van der Meer M, Volkova AY, Frings-Dresen MHW. Illness perceptions and work participation: a systematic review. Int Arch Occup Environ Health. 2010;83:595–605. 10.1007/s00420-010-0506-6. 20130906 10.1007/s00420-010-0506-6 PMC2902734

[30] Giri P, Poole J, Nightingale P, Robertson A. Perceptions of illness and their impact on sickness absence. Occup Med (Lond). 2009;59(8):550–5. 10.1093/occmed/kqp123. 19704030 10.1093/occmed/kqp123

[31] Aakvik A, Holmås TH, Islam MK. Does variation in general practitioner (GP) practice matter for the length of sick leave? A multilevel analysis based on Norwegian GP–patient data. Soc Sci Med. 2010;70(10):1590–8. 10.1016/j.socscimed.2010.01.031. 20226581 10.1016/j.socscimed.2010.01.031

[32] Coudeyre E, Rannou F, Tubach F, Baron G, Coriat F, Brin S, et al. General practitioners’ fear-avoidance beliefs influence their management of patients with low back pain. Pain. 2006;124(3):330–7. 10.1016/j.pain.2006.05.003. 16750297 10.1016/j.pain.2006.05.003

[33] Sanatkar S, Stevelink SAM, Aars NAP, Bardal I, Brandseth OL, Brinchmann B, Mykletun A. Health consequences of graded, full, and no sickness absence among workers with musculoskeletal disorders: health profiles and six-month symptom changes of patients referred to Norwegian outpatient clinics for chronic neck and back pain. BMC Musculoskelet Disord. 2025;26:432. 10.1186/s12891-025-08570-7. 40312295 10.1186/s12891-025-08570-7 PMC12044821

[34] Vlaeyen JW, Linton SJ. Fear-avoidance and its consequences in chronic musculoskeletal pain: a state of the Art. Pain. 2000;85(3):317–32. 10.1016/S0304-3959(99)00242-0. 10781906 10.1016/S0304-3959(99)00242-0

[35] Hagger MS, Orbell S. The common sense model of illness self-regulation: a conceptual review and proposed extended model. Health Psychol Rev. 2021;16(3):347–77. 10.1080/17437199.2021.1878050. 33461402 10.1080/17437199.2021.1878050

[36] Wertli MM, Rasmussen-Barr E, Weiser S, Bachmann LM, Brunner F. The role of fear-avoidance beliefs as a prognostic factor for outcome in patients with nonspecific low back pain: a systematic review. Spine J. 2014;14(5):816–36. 10.1016/j.spinee.2013.09.036. 24412032 10.1016/j.spinee.2013.09.036

[37] Wertli MM, Rasmussen-Barr E, Held U, Weiser S, Bachmann LM, Brunner F. Fear-avoidance beliefs—a moderator of treatment efficacy in patients with low back pain: a systematic review. Spine J. 2014;14(11):2658–78. 10.1016/j.spinee.2014.02.033. 24614254 10.1016/j.spinee.2014.02.033

[38] Joosen MCW, Lugtenberg M, Arends I, van Gestel HJAWM, Schaapveld B, Terluin B, et al. Barriers and facilitators for return to work from the perspective of workers with common mental disorders with short-, medium- and long-term sickness absence: a longitudinal qualitative study. J Occup Rehabil. 2022;32:272–83. 10.1007/s10926-021-10004-9. 34580811 10.1007/s10926-021-10004-9 PMC9232415

[39] Audhoe SS, Nieuwenhuijsen K, Hoving JL, Sluiter JK, Frings-Dresen MHW. Perspectives of unemployed workers with mental health problems: barriers to and solutions for return to work. Disabil Rehabil. 2018;40(1):28–34. 10.1080/09638288.2016.1242170. 27756177 10.1080/09638288.2016.1242170

[40] Noordik E, Nieuwenhuijsen K, Varekamp I, van der Klink JJL, van Dijk FJH. Exploring the return-to-work process for workers partially returned to work and partially on long-term sick leave due to common mental disorders: a qualitative study. Disabil Rehabil. 2011;33(17–18):1625–35. 10.3109/09638288.2010.541547. 21171843 10.3109/09638288.2010.541547

[41] Nigatu YT, Liu Y, Uppal M, McKinney S, Gillis K, Rao S, Wang JL. Prognostic factors for return to work of employees with common mental disorders: a meta-analysis of cohort studies. Soc Psychiatry Psychiatr Epidemiol. 2017;52:1025–1015. 10.1007/s00127-017-1402-0. 10.1007/s00127-017-1402-0 28577211

[42] Villotti P, Kordsmeyer AC, Roy JS, Corbière M, Negrini A, Larivière C. Systematic review and tools appraisal of prognostic factors of return to work in workers on sick leave due to musculoskeletal and common mental disorders. PLoS ONE. 2024;19(7):e0307284. 10.1371/journal.pone.0307284. 39018306 10.1371/journal.pone.0307284 PMC11253986

[43] Lever I, Dyball D, Greenberg N, Stevelink SAM. Health consequences of bullying in the healthcare workplace: a systematic review. J Adv Nurs. 2019;75(12):3195–209. 10.1111/jan.13986. 30816567 10.1111/jan.13986

[44] Nybergh L, Bergström G, Hellman T. Do work- and home-related demands and resources differ between women and men during return-to-work? A focus group study among employees with common mental disorders. BMC Public Health. 2020;20:1914. 10.1186/s12889-020-10045-4. 33334324 10.1186/s12889-020-10045-4 PMC7745371

[45] Villotti P, Gragnano A, Larivière C, Negrini A, Dionne CE, Corbière M. Tools appraisal of organizational factors associated with return-to-work in workers on sick leave due to musculoskeletal and common mental disorders: a systematic search and review. J Occup Rehabil. 2021;31:7–25. 10.1007/s10926-020-09902-1. 32440855 10.1007/s10926-020-09902-1

[46] Blonk RWB, Brenninkmeijer V, Lagerveld SE, Houtman ILD. Return to work: a comparison of two cognitive behavioural interventions in cases of work-related psychological complaints among the self-employed. Work Stress. 2006;20(2):129–44. 10.1080/02678370600856615.

[47] de Vries HJ, Snippen NC, Roelen CAM, et al. Interpersonal processes in the duration of sick leave of workers with chronic diseases: a dyadic analysis. J Occup Rehabil. 2025;35:654–64. 10.1007/s10926-024-10233-8. 39223399 10.1007/s10926-024-10233-8 PMC12361268

[48] Hoff EH, Kraft KB, Moe CF, Nylenna M, Østby KA, Mykletun A. The cost of saying no: general practitioners’ gatekeeping role in sickness absence certification. BMC Public Health. 2024;24:439. 10.1186/s12889-024-17993-1. 38347474 10.1186/s12889-024-17993-1 PMC10860288

[49] OECD. Mental health and work: Norway. Paris: OECD Publishing; 2013. [cited 2025 Dec 28]. 10.1787/9789264178984-en.

[50] Bardal I, Aars NAP, Trichet LO, Brandseth OL, Terjesen C, Irgens E, et al. Expanding the focus on work factors in an outpatient setting: does a nudge of patients and clinicians have an effect on return-to-work and benefits? Findings from the NSAC nudge multicentre randomised controlled trial. J Occup Rehabil. 2025. 10.1007/s10926-025-10343-x.41385138 10.1007/s10926-025-10343-x

