# Supplementary material for: Barriers for return to work as an iatrogenic effect of sickness absence: a proposed conceptual framework and questionnaire based on a cross-sectional study
Source: BMC Public Health. 2026 Feb 12;26:920. doi: 10.1186/s12889-026-26584-1 (PMC12998097; doi:10.1186/s12889-026-26584-1)
Supplement: Supplementary file 1 — Additional file 1. Response distribution to barrier items. Overview of barrier items, with response distribution, histogram, mean and standard deviation of each item. [file 12889_2026_26584_MOESM1_ESM.pdf]

Additional file 1 Response distribution to barrier items

There can be many barriers that prevent you from returning to work when you are on sick leave. How do you perceive the following statements?	Response distribution N (%)	Histogram<br>1: Disagree<br>5: Agree	Mean	St.d.
I can't go back to work now because I'm responsible for the care of a friend or family member (family)	| | N | % | | 1 | 233 | 83,8% | | 2 | 17 | 6,1% | | 3 | 16 | 5,8% | | 4 | 6 | 2,2% | | 5 | 6 | 2,2% |	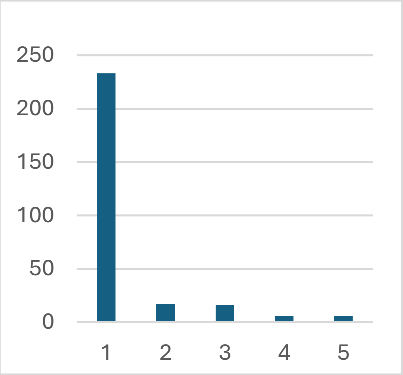	1.33	0.85
I can't go back to work now because I'm in a stressful life situation (for example, illness of others, breakup, conflict or death in my own family) (family)	| | N | % | | 1 | 214 | 77,0% | | 2 | 15 | 5,4% | | 3 | 23 | 8,3% | | 4 | 8 | 2,9% | | 5 | 18 | 6,5% |	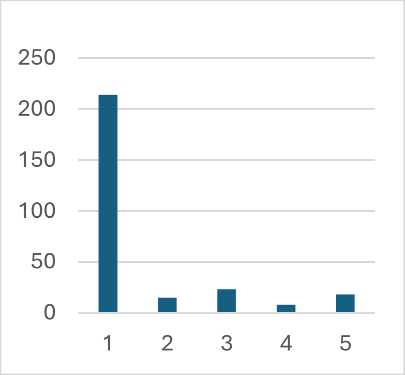	1.56	1.17

		N	%			
I can’t go back to work now because I don’t have enough time (family)		N	%	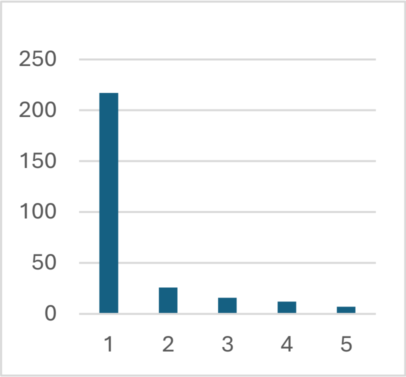	1.44	0.96
	1	217	78,1%			
	2	26	9,4%			
	3	16	5,8%			
	4	12	4,3%			
	5	7	2,5%			
I can’t go back to work now because I don’t feel ready to start working yet (emotions)		N	%	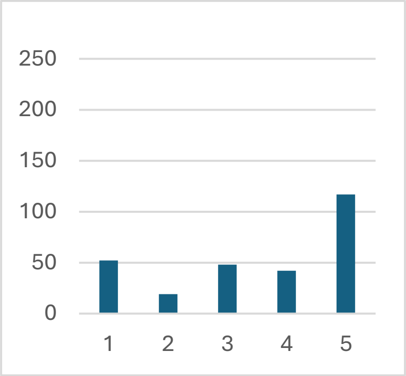	3.55	1.54
	1	52	18,7%			
	2	19	6,8%			
	3	48	17,3%			
	4	42	15,1%			
	5	117	42,1%			
I can’t go back to work now because I don’t master my job (emotions)		N	%	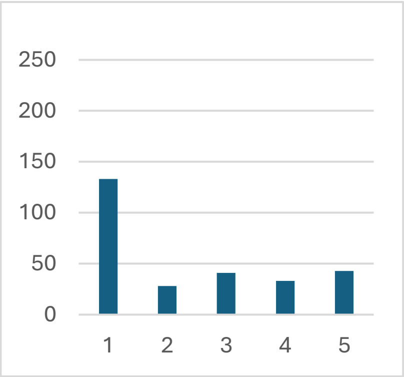	2.37	1.54
	1	133	47,8%			
	2	28	10,1%			
	3	41	14,7%			
	4	33	11,9%			
	5	43	15,5%			

Item		N	%	Chart	Mean	SD
I can't go back to work now because I'm not motivated for the job I have now (emotions)	1	164	59,0%	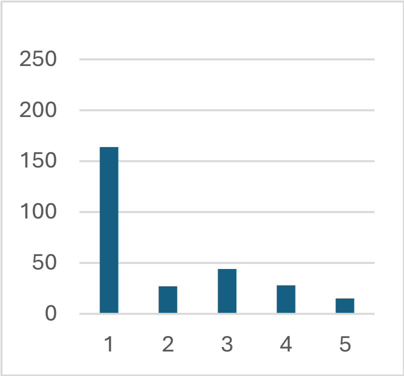	1.93	1.28
	2	27	9,7%			
	3	44	15,8%			
	4	28	10,1%			
	5	15	5,4%			
I can't go back to work now because I have to set boundaries for myself (emotions)	1	66	23,7%	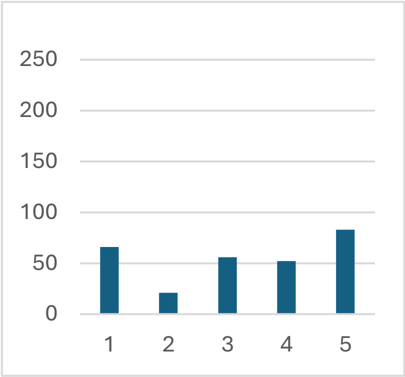	3.23	1.54
	2	21	7,6%			
	3	56	20,1%			
	4	52	18,7%			
	5	83	29,9%			
I can't go back to work now because I can then be laid off, downsized or relocated (emotions)	1	247	88,8%	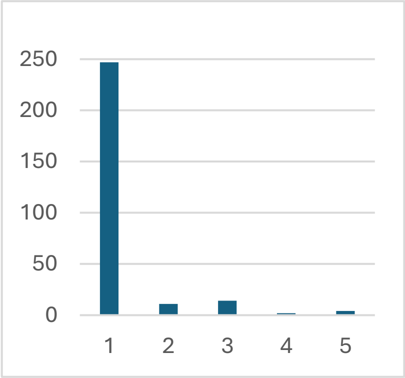	1.22	0.70
	2	11	4,0%			
	3	14	5,0%			
	4	2	0,7%			
	5	4	1,4%			
I can't go back to work now because I have to get well first (cognitions)	1	26	9,4%	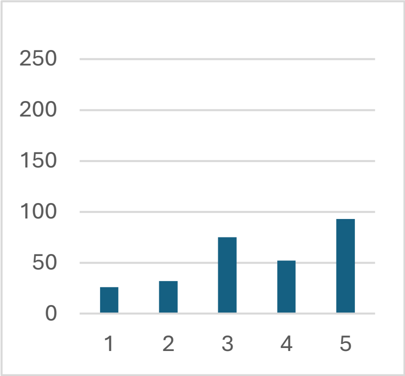	3.55	1.31
	2	32	11,5%			
	3	75	27,0%			
	4	52	18,7%			
	5	93	33,5%			

		N	%			
I can’t go back to work now because I can get sicker if I go back to work (cognitions)	1	20	7,2%	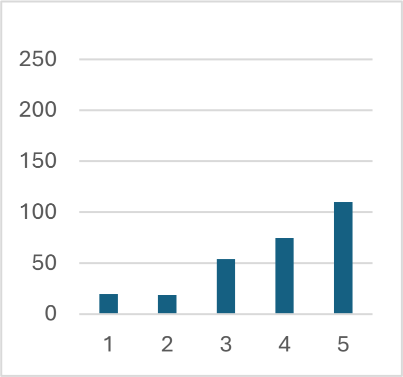	3.85	1.22
	2	19	6,8%			
	3	54	19,4%			
	4	75	27,0%			
	5	110	39,6%			
I can’t go back to work now because my doctor thinks I should be on sick leave now (cognitions)	1	55	19,8%	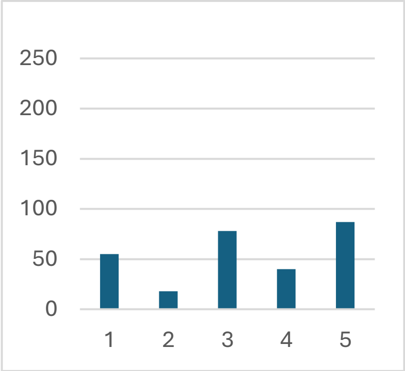	3.31	1.47
	2	18	6,5%			
	3	78	28,1%			
	4	40	14,4%			
	5	87	31,3%			
I can’t go back to work now because I have to focus on treatment (cognitions)	1	32	11,5%	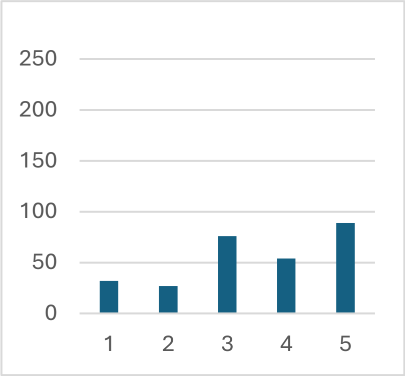	3.51	1.33
	2	27	9,7%			
	3	76	27,3%			
	4	54	19,4%			
	5	89	32,0%			
I can’t go back to work now because I have too much physical pain (assessment)	1	54	19,4%	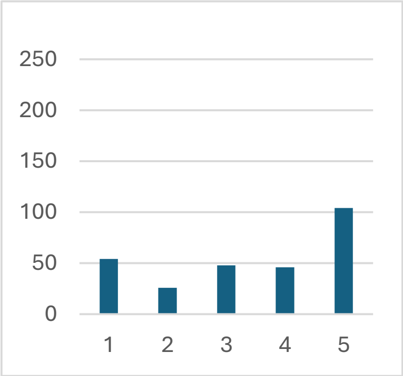	3.43	1.54
	2	26	9,4%			
	3	48	17,3%			
	4	46	16,5%			
	5	104	37,4%			

<table>
<tr>
<td>I can’t go back to work now because I have concentration problems (assessment)</td>
<td></td>
<td><table><tr><td></td><td>N</td><td>%</td></tr><tr><td>1</td><td>72</td><td>25,9%</td></tr><tr><td>2</td><td>35</td><td>12,6%</td></tr><tr><td>3</td><td>67</td><td>24,1%</td></tr><tr><td>4</td><td>56</td><td>20,1%</td></tr><tr><td>5</td><td>48</td><td>17,3%</td></tr></table></td>
<td></td>
<td>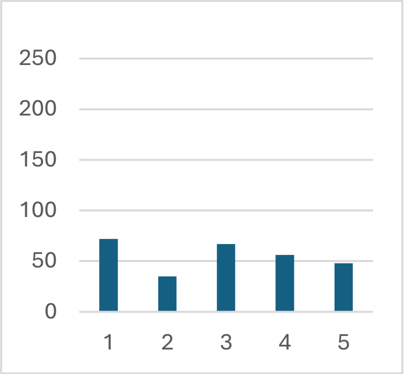
</td>
<td>2.90</td>
<td>1.43</td>
</tr>
<tr>
<td>I can’t go back to work now because I’m too anxious or scared (assessment)</td>
<td></td>
<td><table><tr><td></td><td>N</td><td>%</td></tr><tr><td>1</td><td>162</td><td>58,3%</td></tr><tr><td>2</td><td>35</td><td>12,6%</td></tr><tr><td>3</td><td>36</td><td>12,9%</td></tr><tr><td>4</td><td>32</td><td>11,5%</td></tr><tr><td>5</td><td>13</td><td>4,7%</td></tr></table></td>
<td></td>
<td>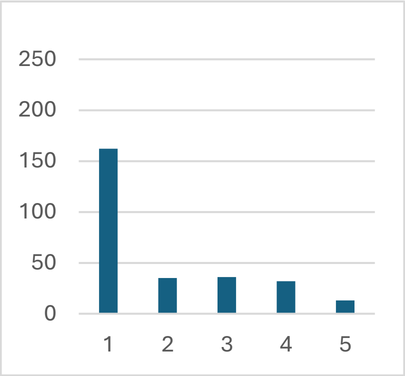
</td>
<td>1.92</td>
<td>1.26</td>
</tr>
<tr>
<td>I can’t go back to work now because I’m too depressed (assessment)</td>
<td></td>
<td><table><tr><td></td><td>N</td><td>%</td></tr><tr><td>1</td><td>143</td><td>51,4%</td></tr><tr><td>2</td><td>32</td><td>11,5%</td></tr><tr><td>3</td><td>40</td><td>14,4%</td></tr><tr><td>4</td><td>43</td><td>15,5%</td></tr><tr><td>5</td><td>20</td><td>7,2%</td></tr></table></td>
<td></td>
<td>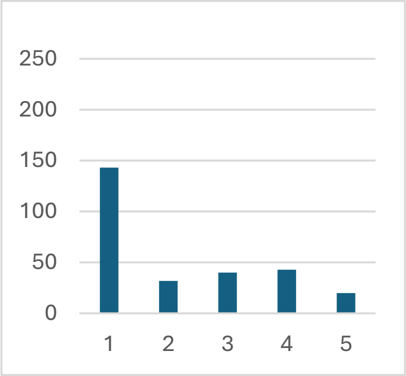
</td>
<td>2.15</td>
<td>1.38</td>
</tr>
</table>

<table>
<tr>
<td>I can’t go back to work now because I do not have enough energy (assessment)</td>
<td>
<table>
<tr><th></th><th>N</th><th>%</th></tr>
<tr><td>1</td><td>42</td><td>15,1%</td></tr>
<tr><td>2</td><td>14</td><td>5,0%</td></tr>
<tr><td>3</td><td>45</td><td>16,2%</td></tr>
<tr><td>4</td><td>75</td><td>27,0%</td></tr>
<tr><td>5</td><td>102</td><td>36,7%</td></tr>
</table>
</td>
<td>
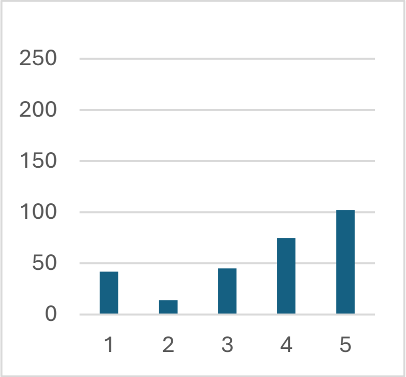

</td>
<td>3.65</td>
<td>1.41</td>
</tr>
<tr>
<td>I can’t go back to work now because I have too much sleep problems (assessment)</td>
<td>
<table>
<tr><th></th><th>N</th><th>%</th></tr>
<tr><td>1</td><td>80</td><td>28,8%</td></tr>
<tr><td>2</td><td>47</td><td>16,9%</td></tr>
<tr><td>3</td><td>56</td><td>20,1%</td></tr>
<tr><td>4</td><td>52</td><td>18,7%</td></tr>
<tr><td>5</td><td>43</td><td>15,5%</td></tr>
</table>
</td>
<td>
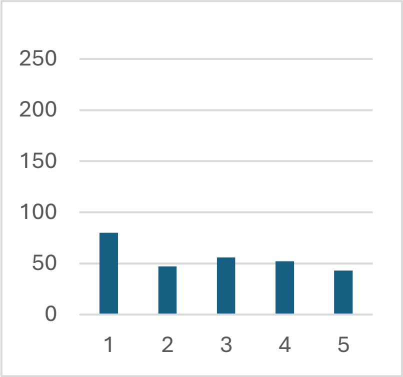

</td>
<td>2.75</td>
<td>1.44</td>
</tr>
<tr>
<td>I can’t go back to work now because my employer does not adapt tasks to my situation (work)</td>
<td>
<table>
<tr><th></th><th>N</th><th>%</th></tr>
<tr><td>1</td><td>145</td><td>52,2%</td></tr>
<tr><td>2</td><td>38</td><td>13,7%</td></tr>
<tr><td>3</td><td>59</td><td>21,2%</td></tr>
<tr><td>4</td><td>14</td><td>5,0%</td></tr>
<tr><td>5</td><td>22</td><td>7,9%</td></tr>
</table>
</td>
<td>
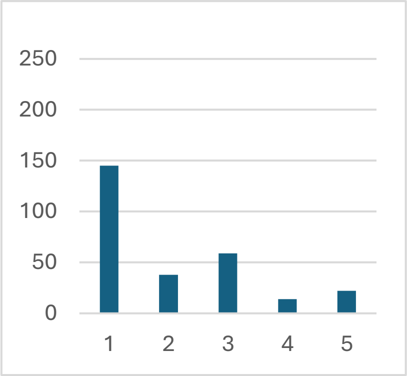

</td>
<td>2.03</td>
<td>1.29</td>
</tr>
</table>

		N	%			
I can’t go back to work now because I am being treated badly at work (work)		N	%	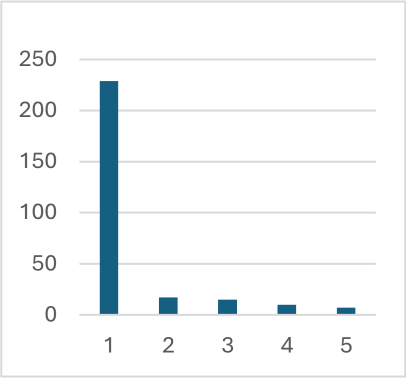	1.38	0.93
	1	229	82,4%			
	2	17	6,1%			
	3	15	5,4%			
	4	10	3,6%			
	5	7	2,5%			
I can’t go back to work now because I am not wanted at work (work)		N	%	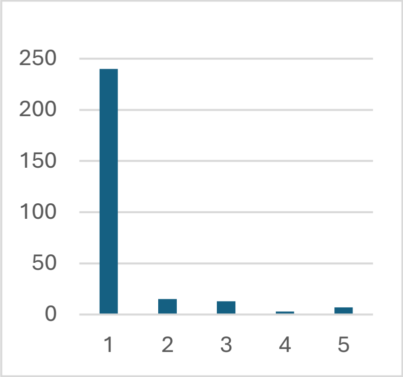	1.28	0.82
	1	240	86,3%			
	2	15	5,4%			
	3	13	4,7%			
	4	3	1,1%			
	5	7	2,5%			
I can’t go back to work now because I’m in a conflict at work (work)		N	%	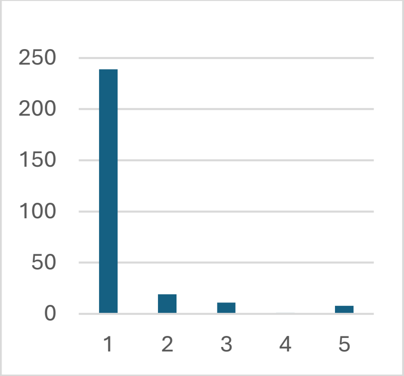	1.27	0.80
	1	239	86,0%			
	2	19	6,8%			
	3	11	4,0%			
	4	1	0,4%			
	5	8	2,9%			
I can’t go back to work now because I’m waiting for an apology (work)		N	%	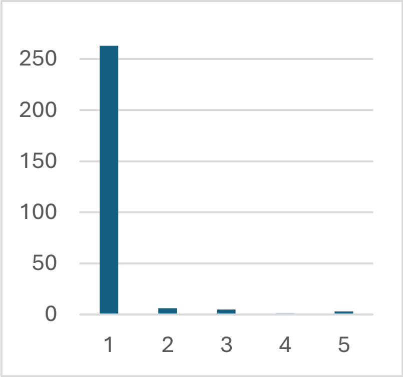	1.11	0.54
	1	263	94,6%			
	2	6	2,2%			
	3	5	1,8%			
	4	1	0,4%			
	5	3	1,1%			

I can't go back to work now because I' m trying to send a signal to management or the business (work)		| N | % | 1 | 249 | 89,6%<br>2 | 15 | 5,4%<br>3 | 9 | 3,2%<br>4 | 2 | 0,7%<br>5 | 3 | 1,1%		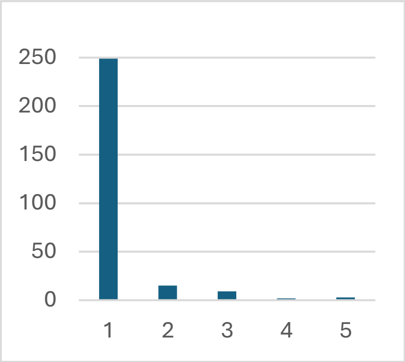	1.18	0.62
I can't go back to work now because something at work has changed while I have been on sick leave (work)		| N | % | 1 | 249 | 89,6%<br>2 | 13 | 4,7%<br>3 | 7 | 2,5%<br>4 | 3 | 1,1%<br>5 | 6 | 2,2%		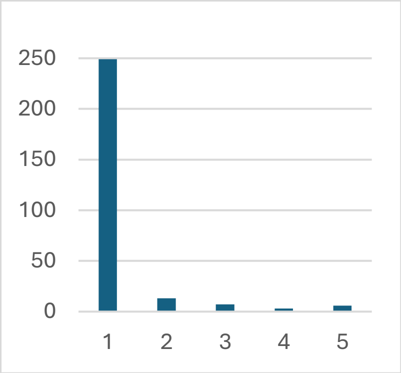	1.22	0.74
I can't go back to work now because the job makes me sick (work)		| N | % | 1 | 115 | 41,4%<br>2 | 40 | 14,4%<br>3 | 52 | 18,7%<br>4 | 42 | 15,1%<br>5 | 29 | 10,4%		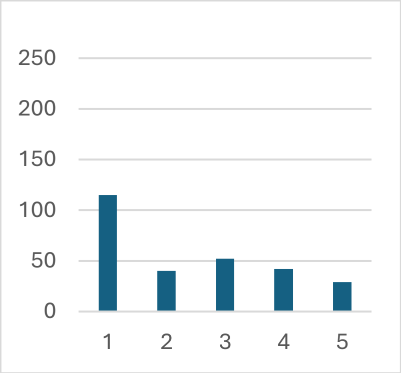	2.39	1.41

The text in parenthesis is to indicate the subgroup in which the item is categorised. Family: Family obligations, Emotions: Emotions about RTW, Cognitions: Cognitions about health, Assessment: Assessment of health problems, Work: Work-related factors.
